# Preimplant factors affecting postimplant CT-determined prostate volume and the CT/TRUS volume ratio after transperineal interstitial prostate brachytherapy with ^125^I free seeds

**DOI:** 10.1186/1748-717X-5-86

**Published:** 2010-09-28

**Authors:** Akitomo Sugawara, Jun Nakashima, Etsuo Kunieda, Hirohiko Nagata, Hirotaka Asakura, Mototsugu Oya, Naoyuki Shigematsu

**Affiliations:** 1 Department of Radiology, Keio University School of Medicine, Tokyo, Japan; 2 Department of Urology, Tokyo Medical University, Tokyo, Japan; 3 Department of Radiation Oncology, Tokai University School of Medicine, Isehara, Japan; 4 Department of Urology, Keio University School of Medicine, Tokyo, Japan; 5 Department of Urology, Saitama Medical University, Saitama, Japan

## Abstract

**Background:**

The aim was to identify preimplant factors affecting postimplant prostate volume and the increase in prostate volume after transperineal interstitial prostate brachytherapy with ^125^I free seeds.

**Methods:**

We reviewed the records of 180 patients who underwent prostate brachytherapy with ^125^I free seeds for clinical T1/T2 prostate cancer. Eighty-one (45%) of the 180 patients underwent neoadjuvant hormonal therapy. No patient received supplemental external beam radiotherapy. Postimplant computed tomography was undertaken, and postimplant dosimetric analysis was performed. Univariate and multivariate analyses were performed to identify preimplant factors affecting postimplant prostate volume by computed tomography and the increase in prostate volume after implantation.

**Results:**

Preimplant prostate volume by transrectal ultrasound, serum prostate-specific antigen, number of needles, and number of seeds implanted were significantly correlated with postimplant prostate volume by computed tomography. The increase in prostate volume after implantation was significantly higher in patients with neoadjuvant hormonal therapy than in those without. Preimplant prostate volume by transrectal ultrasound, number of needles, and number of seeds implanted were significantly correlated with the increase in prostate volume after implantation. Stepwise multiple linear regression analysis showed that preimplant prostate volume by transrectal ultrasound and neoadjuvant hormonal therapy were significant independent factors affecting both postimplant prostate volume by computed tomography and the increase in prostate volume after implantation.

**Conclusions:**

The results of the present study show that preimplant prostate volume by transrectal ultrasound and neoadjuvant hormonal therapy are significant preimplant factors affecting both postimplant prostate volume by computed tomography and the increase in prostate volume after implantation.

## Background

As transperineal interstitial prostate brachytherapy becomes more widely used for early localized prostate cancer, there is growing interest in quality assurance measures that include postimplant dosimetric analysis [1-3]. One impediment to meaningful dosimetric analysis is unexpected prostate volume changes due to seed implantation. Mechanical trauma, induction of an inflammatory response, and intraprostatic bleeding are possible mechanisms. Prostatic swelling occurs during and after implantation, and, in general, is the greatest on the day of operation and the following day. Over time, the prostate subsequently decreases in size. About one month after implantation, prostatic swelling is mostly settled. Postimplant computed tomography (CT) is recommended at this time [4]. Sometimes, however, the postimplant prostate volumes at this time are still larger than the preimplant volumes, and the degree varies among patients [5-7]. The variability of volume increase can significantly influence the postimplant dosimetric evaluation [8-10]. To our knowledge, the pathogenesis of the volume change of the prostate and preimplant factors affecting the increase in prostate volume after implantation are not well researched. Identification of the preimplant factors could be useful in preplanning seed placement to compensate for the increase. The present study was undertaken to identify preimplant factors affecting the volume change of the prostate after transperineal interstitial prostate brachytherapy with ^125^I free seeds.

## Methods

We reviewed the records of 180 patients who underwent transperineal interstitial prostate brachytherapy with ^125^I free seeds for clinical T1/T2 prostate cancer at our institution. Table 1 details the characteristics of all 180 patients. One hundred thirty-two (73.3%) patients had a Gleason score of 6 or less and 48 (26.7%) patients had a Gleason score of 7. The mean ± standard error (SE) prostate-specific antigen (PSA) level was 7.06 ± 0.23 ng/mL (range, 4.01-19.88 ng/mL). Eighty-one (45%) of the 180 patients underwent 5.1 ± 0.3 months of neoadjuvant hormonal therapy (NHT), which consisted of luteinizing hormone-releasing hormone agonist and antiandrogens. NHT was generally undertaken in patients with a prostate volume >40 cc or those with pubic arch interference at the preimplant volume study by transrectal ultrasound (TRUS) [11]. Hormonal therapy was not continued past the date of implant.

**Table 1:** Patient characteristics (N = 180)

Variable		Range
Age (y)	68.7 ± 0.5	(53-80)
Initial PSA (ng/mL)	7.06 ± 0.23	(4.01-19.88)
NHT (+), *n *(%)	81 (45.0%)	
NHT (-), *n *(%)	99 (55.0%)	
Preimplant prostate volume by TRUS (cc)	22.9 ± 0.5	(10.1-41.0)
Total radioactivity (mCi)	23.5 ± 0.3	(13.1-33.6)
Number of seeds implanted	70.9 ± 0.9	(40-100)
Number of needles	25.0 ± 0.4	(12-39)

Data are presented as mean ± standard error (range) or number (percent) of patients. *Abbreviations: *PSA = prostate-specific antigen; NHT = neoadjuvant hormonal therapy; TRUS = transrectal ultrasound.

A preplan was obtained using TRUS images taken at 5 mm intervals from the base to the apex of the prostate with the patient in the dorsal lithotomy position at one month before implantation. The prostatic contours were outlined by a single radiation oncologist (AS). The planning target volume included the prostate gland, with a margin of 3 mm anteriorly and laterally and 5 mm in the cranial and caudal directions. No margin was added posteriorly at the rectal interface. Treatment planning used a peripheral or a modified peripheral approach. The prescribed dose to the planning target volume (prostate with margin) was 145 Gy. Preplan dosimetry aimed for a prostate V100 (% of the prostate volume receiving the prescribed dose or greater) of >99%, a prostate D90 (dose to 90% of the prostate) of 120% to 125% of the prescribed dose, a prostate V150 of 55% to 60%, a urethra V100 (% of the urethral volume receiving the prescribed dose or greater) of >99%, a urethra V150 of 0%, and a rectum V100 (cc of the rectum volume receiving the prescribed dose or greater) of < 1.3cc, and a rectum V150 of 0cc. During preplanning with TRUS and seed implantation, the urethra was identified with aerated gel for ease of visualization. An attempt was made during the manual planning to place seeds into the prostate, not to place seeds within 0.5 cm of the urethra. VariSeed 7.1 (Varian Medical Systems, Palo Alto, CA) was used both in planning and in calculation of the final dosimetry. TG 43 formalism was used in the preplanning and postimplant dosimetry analyses [12]. All 180 patients were treated with ^125^I radioactive free seeds with a Mick applicator (Mick Radio-Nuclear Instruments, Bronx, NY). The radioactive seeds were inserted according to the preplan, under TRUS and fluoroscopic guidance. No supplemental external beam radiotherapy was used. Postimplant axial CT images of the prostate at 2.5 to 3.0-mm intervals with patients in the supine position were obtained at a mean ± SE of 7.6 ± 0.2 weeks after implantation. The prostatic contours were outlined by a single radiation oncologist (AS). The postimplant prostatic margins were similar to preplan margins. Postimplant dosimetry calculations were performed. The following information was recorded: patient characteristics, preimplant prostate volume by TRUS, postimplant prostate volume by CT, and postimplant CT scan volume relative to the ratio of the preimplant TRUS volume of the prostate (CT/TRUS volume ratio).

### Statistical analysis

Data are presented as mean ± SE. Pearson correlation coefficients were used to examine the relationship between postimplant prostate volume by CT and continuous variables and that between the CT/TRUS volume ratio and continuous variables. Associations between categorical variables were assessed with Fisher's exact test. Student's t-test was used for quantitative data. The significance level was *p *< 0.05.

Stepwise multiple linear regression analyses were performed to estimate postimplant prostate volume by CT and the CT/TRUS volume ratio from age, serum PSA, NHT, preimplant prostate volume by TRUS, number of needles, and number of seeds implanted.

## Results

The mean ± SE preimplant and postimplant D90 were 176.6 ± 1.0 Gy and 171.4 ± 1.5 Gy, respectively. The mean ± SE preimplant and postimplant V100 were 97.2 ± 0.2% and 95.4 ± 0.3%, respectively. The mean ± SE postimplant prostate volume by CT was 26.0 ± 0.5 cc (range, 10.8 - 51.0 cc). Postimplant prostate volume by CT was not significantly different between patients with NHT and those without (25.9cc vs. 26.1cc, *p *= 0.769). The mean ± SE postimplant D90 in patients with NHT and those without were 166.5 ± 1.9Gy (range, 123.3 - 223.0Gy) and 175.4 ± 2.2Gy (range, 125.9 - 223.6Gy), respectively. The mean ± SE postimplant V100 in patients with NHT and those without were 94.9 ± 0.4% (range, 76.5 - 99.9%) and 95.8 ± 0.4% (range, 83.2 - 100.0%), respectively. The mean ± SE number of seeds implanted in patients with NHT and those without were 67.2 ± 1.3 (range, 40 - 95) vs. 73.9 ± 1.2 (range, 45 - 100), respectively. The mean ± SE number of needles in patients with NHT and those without were 22.6 ± 0.6 (range, 12 - 37) vs. 26.9 ± 0.5 (range, 15 - 39), respectively. Preimplant prostate volume by TRUS (r = 0.802, *p *< 0.001), serum PSA (r = 0.159, *p *= 0.034), number of needles (r = 0.582, *p *< 0.001), and number of seeds implanted (r = 0.760, *p *< 0.001) were significantly and positively correlated with postimplant prostate volume by CT.

**Figure 1:**
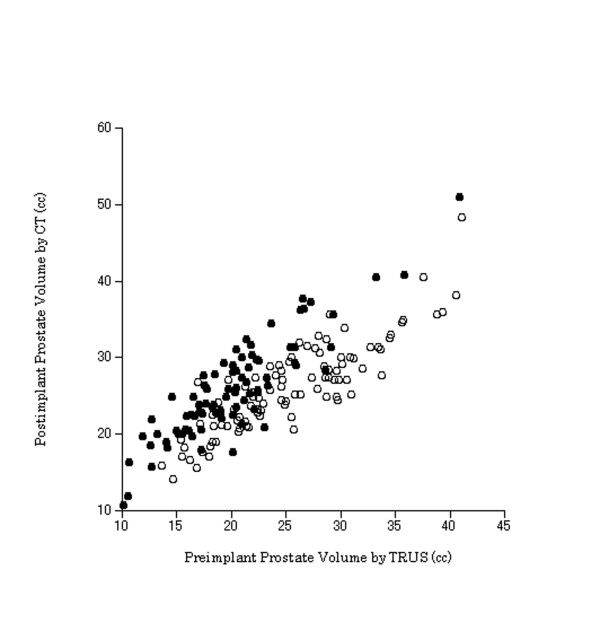
**Postimplant CT vs. preimplant TRUS volume of the prostate for patients with NHT (black circle) and those without (blank circle)**. Data are shown for all 180 patients. Abbreviations: CT = computed tomography; TRUS = transrectal ultrasound; NHT = neoadjuvant hormonal therapy.

Stepwise multiple linear regression analysis showed that preimplant prostate volume by TRUS and NHT were significant independent factors affecting postimplant prostate volume by CT (Table 2). The predictive equation for postimplant prostate volume by CT (cc) was as follows: V = a1 + a2Vp +a3x, where V = postimplant prostate volume, Vp = preimplant prostate volume by TRUS and x = 1 or 0 for with or without NHT. The values of a1, a2 and a3 were 3.390, 0.899, and 4.427, respectively. Figure 1 shows the plot of the postimplant CT vs. the preimplant TRUS volume of the prostate for patients with NHT and those without.

**Table 2:** Multivariate analysis: stepwise multiple linear regression model for postimplant prostate volume by CT

Covariate	B	SE	*p *value
Constant	3.390	1.031	0.001
Preimplant prostate volume by TRUS (cc)	0.899	0.039	< 0.001
NHT	4.427	0.503	< 0.001

*Abbreviations: *CT = computed tomography; B: unstandardized coefficients; SE: standard errors of unstandardized coefficients; TRUS = transrectal ultrasound; NHT = neoadjuvant hormonal therapy.

The mean ± SE CT/TRUS volume ratio was 1.16 ± 0.01 (range, 0.80 - 1.74). The CT/TRUS volume ratio was significantly higher in patients with NHT than in those without (1.30 vs. 1.05, *p *< 0.001). Preimplant prostate volume by TRUS (r = -0.538, *p *< 0.001), number of needles (r = -0.505, *p *< 0.001), and number of seeds implanted (r = -0.408, *p *< 0.001) were significantly and negatively correlated with the CT/TRUS volume ratio.

**Figure 2:**
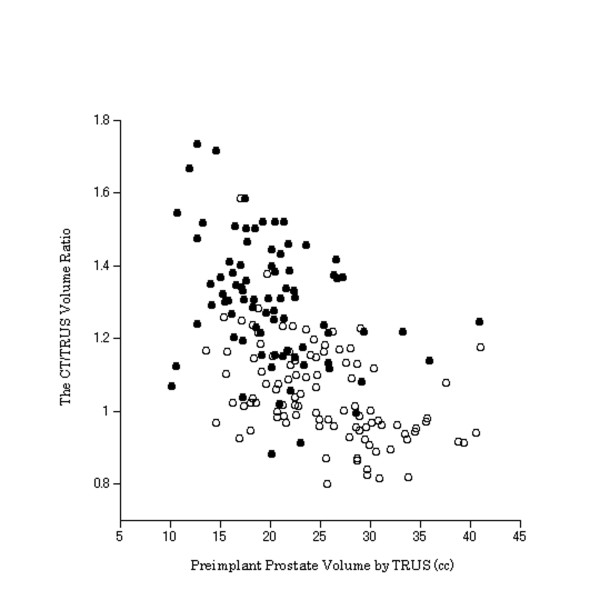
**The CT/TRUS volume ratio vs. preimplant prostate volume by TRUS for patients with NHT (black circle) and those without (blank circle)**. Data are shown for all 180 patients. *Abbreviations*: CT = computed tomography; TRUS = transrectal ultrasound; the CT/TRUS volume ratio = the postimplant CT scan volume relative to the ratio of the preimplant TRUS volume of the prostate; NHT = neoadjuvant hormonal therapy.

Stepwise multiple linear regression analysis showed that preimplant prostate volume by TRUS and NHT were significant independent factors affecting the CT/TRUS volume ratio (Table 3). The predictive equation of the CT/TRUS volume ratio was as follows: the CT/TRUS volume ratio = b1 + b2Vp + b3×, where Vp = preimplant prostate volume by TRUS and × = 1 or 0 for with or without NHT. The values of b1, b2 and b3 were 1.303, - 0.010, and 0.204, respectively. Figure 2 shows the plot of the CT/TRUS volume ratio vs. the preimplant prostate volume by TRUS for patients with NHT and those without.

**Table 3:** Multivariate analysis: stepwise multiple linear regression model for the CT/TRUS volume ratio

Covariate	B	SE	*p *value
Constant	1.303	0.047	< 0.001
Preimplant prostate volume by TRUS (cc)	-0.010	0.002	< 0.001
NHT	0.204	0.023	< 0.001

*Abbreviations: *CT = computed tomography; TRUS = transrectal ultrasound; CT/TRUS volume ratio = postimplant CT scan volume relative to the ratio of the preimplant TRUS volume of the prostate; B: unstandardized coefficients; SE: standard errors of unstandardized coefficients; NHT = neoadjuvant hormonal therapy.

## Discussion

The purpose of the present study was to identify preimplant factors affecting postimplant CT-determined prostate volume and the CT/TRUS volume ratio in prostate cancer patients treated with transperineal interstitial prostate brachytherapy with ^125^I free seeds. The results showed that preimplant prostate volume by TRUS and NHT are significant independent factors affecting both postimplant prostate volume by CT and the CT/TRUS volume ratio. From these results, we have developed regression equations to predict postimplant prostate volume by CT and the CT/TRUS volume ratio.

NHT was revealed to be a significant preimplant factor affecting both postimplant prostate volume by CT and the increase in prostate volume after implantation. The present results indicate that a patient with NHT has a higher CT/TRUS volume ratio than a patient without. These results are in agreement with a previous report [13]. Ash and coworkers reported that the mean CT/TRUS volume ratio was 1.17 for patients with NHT vs. 0.98 for those without (*p *< 0.001) [13]. This means that patients with NHT have a greater increase in prostate volume after implantation. The reason why NHT is positively associated with the increase in prostate volume after implantation is unclear. It has been reported that NHT is associated with an increased risk of acute urinary morbidity after implantation [14-17]. The association of NHT with the increase in prostate volume would provide an explanation for this clinical observation, however, it does not explain the pathophysiology of the increased tendency to prostate swellings. This issue needs to be explored in further studies.

The present results, however, may seem to contradict those of some other studies [18-20]. Badiozamani and colleagues reported that NHT had no consistent effect on postimplant volume changes [18]. Tanaka and colleagues reported that no predictive factors for edema were found, including NHT [20]. Potters and colleagues reported that the CT/TRUS volume ratio was significantly lower for patients treated with NHT [19]. The discrepancies could be attributed to differences in the timing of postimplant CT scans among these studies. In the studies by Badiozamani and colleagues and Tanaka and colleagues, the postimplant CT scans were obtained the day after implantation, when prostatic swelling was the greatest. In the study by Potters and colleagues, they were obtained 1.6-6.5 weeks (median, 3.1 weeks) after implantation, which seems somewhat early for all of the prostatic edema to resolve, whereas, in the study by Ash and coworkers, they were obtained 6-8 weeks after implantation, when the swelling had resolved [8,19]. These findings suggest that the impact of NHT on postimplant prostate volume changes differs depending on the timing of the postimplant CT scans. In the present study, the postimplant CT scans were obtained at a mean of 7.6 weeks after implantation, which is similar to the study of Ash and coworkers. Consequently, it is considered that the results of the present study are consistent with those by Ash and coworkers, showing a positive association between the CT/TRUS volume ratio and NHT. However, further study will be needed to assess the potential impact of NHT on the increase in prostate volume after implantation.

The next factor affecting postimplant prostate volume by CT and the increase in prostate volume after implantation is preimplant prostate volume by TRUS. In univariate and multivariate analyses, preimplant prostate volume by TRUS was associated significantly with postimplant prostate volume by CT and the CT/TRUS volume ratio. The present results indicate that a patient with a smaller gland has a higher CT/TRUS volume ratio. This is consistent with a previous report [21]. Pinkawa and colleagues reported that preimplant prostate volume was correlated with the extent of postimplant edema both on day 1 and day 30, indicating that smaller prostates developed greater edema [21]. However, some previous reports are inconsistent with the present study [6,18]. Badiozamani and colleagues reported that no single parameter, including preimplant prostate volume, could accurately predict the degree of swelling on day 1 [18]. Moreover, Taussky and colleagues reported in their study consisting of only 20 patients that, although preimplant prostate volume was associated with the CT/TRUS volume ratio on Day 1, it was not associated with the CT/TRUS volume ratio on Day 30 [6]. These discrepancies are due to the different timings of postimplant CT scans and the small numbers of patients in their studies. Further study will be needed to assess the potential impact of preimplant prostate volume on the volume increases after implantation.

Although the CT/TRUS volume ratio is important because it affects postimplant dosimetric results, it has not been fully determined what the optimal cut-off value of the CT/TRUS volume ratio should be for predicting suboptimal dosimetry. Few investigators have determined a cut-off value of the CT/TRUS volume ratio for predicting suboptimal dosimetry [19]. Potters and colleagues reported that a CT/TRUS volume ratio >1.5 was an independent predictor of poor D90 dose [19]. It is, however, difficult to compare their results directly to ours. The reasons are as follows. First, the results of Potters and colleagues showed a relatively higher CT/TRUS volume ratio (mean, 1.43), due to the early timing of postimplant CT scans. On the contrary, the results of the present study showed the mean CT/TRUS volume ratio of 1.16, which is in agreement with those of many other studies [6,7,13,19,22,23]. Second, in the Cox regression analysis of Potters and colleagues, which was performed to identify independent factors predictive of poor D90 dose, patients with NHT were excluded [19]. Therefore, the cut-off value presented by Potters and colleagues could not be applied directly to the present data. An optimal cut-off value of the CT/TRUS volume ratio to predict suboptimal dosimetry will need to be explored in further studies.

The results of the present study show that in patients who had a smaller prostate gland and/or who underwent NHT, a greater increase in prostate volume is predicted after brachytherapy, which may affect postimplant dosimetric results. For these patients, to achieve optimal dose coverage of the prostate, it is thought to be useful to implant more seeds than expected. However, this speculation should be validated in future investigations.

## Conclusions

The results of the present study show that NHT and preimplant prostate volume by TRUS are significant preimplant factors affecting both postimplant prostate volume by CT and the CT/TRUS volume ratio. Thus, the combination of these two factors can be used to predict postimplant prostate volume by CT and the CT/TRUS volume ratio in prostate cancer patients treated with transperineal interstitial prostate brachytherapy with ^125^I free seeds.

## Competing interests

The authors declare that they have no competing interests.

## Authors' contributions

AS and JN designed the study, collected the data, interpreted the results of the study, performed the statistical analysis and drafted the manuscript, and oversaw the project completion. EK, HN, and HA participated in preparing of data acquisition. MO and NS contributed to data analysis. All authors read and approved the manuscript.
